# Supplementary material for: Patterns of Intron Gain and Loss in Fungi
Source: PLoS Biol. 2004 Nov 30;2(12):e422. doi: 10.1371/journal.pbio.0020422 (PMC532390; doi:10.1371/journal.pbio.0020422)
Supplement: Table S1 — Also available at http://genes.mit.edu/NielsenEtAl/. (4.3 MB ZIP). [file pbio.0020422.st001.zip › NielsenEtAl/html/1251.html]

AN2229.1.NCU07001.1.MG01469.1.FG05658.1


```
 CLUSTAL W (1.82) Multiple Sequence Alignments - Introns Inserted


Sequence 1: NCU07001.1	498 aa
Sequence 2: MG01469.1	504 aa
Sequence 3: FG05658.1	502 aa
Sequence 4: AN2229.1	489 aa
Alignment Length: 532 aa
Number Identitical Residues: 308 aa
Alignment Score (without introns) 13807


MG01469.1 	MSSSSPTGGERL----~-------------RYE1R~TRSQPENPFATLIPDQQIAIIPSF
NCU07001.1	MSNTTTA---------~---------------E1R~IQSQPENPFAALIPDQQIAVIPSF
FG05658.1 	MSEINTTNGDSRKHYA1PYTVILYITVYHNGSP~I2TQSQPENPFAALIPEQQLAIVPEF
AN2229.1  	MTTEQPTAR-------~--------------LQ1R~VDSQPENPFSALIEDQSIVIIPTF
          	*:   .:                               *******::** :*.:.::* *

MG01469.1 	TLESGVTLRNVPVAYTTRGQLNAAGDNAMVICHALTGSADVADWWGPLLGGPGRAFDTSR
NCU07001.1	TLESGVTLHNVPVAYTTRGTLNADGDNAMVICHALTGSADVSDWWGPLLGGPGRAFDVSR
FG05658.1 	TLESGVTLYNLPVAYTTRGKLNEEGNNAMVICHALTGSADVSDWWGPLLGGPGRAFDTSR
AN2229.1  	TLESGVTLYNVPVAYTTRGTLSPSGDNALVICHALSGSADVADWWGPLLGGPGQAFDISR
          	******** *:******** *.  *:**:******:*****:***********:*** **

MG01469.1 	FFIVCMNSLGSPYGTASPVTAKDGDPSLGQYGPEFPLTTIRDDVN2LHKMLLDDLGVKQV
NCU07001.1	FFIVCFNSLGSPYGTASPVTAKDGDPTKGRYGPEFPLTTIRDDVR~LHKLLLDDLGVRQV
FG05658.1 	FFIVCMNSLGSPYGTASPVTAKDNDPSKGRYGPEFPLTTIRDDVN2LHKLILDDLGVKQL
AN2229.1  	FFVVCLNSLGSPYGSASAVTYKDGNPEKGLYGPEFPLTTVRDDVR2IHKMVLDDLGIKQI
          	**:**:********:**.** **.:*  * *********:****. :**::*****::*:

MG01469.1 	AAVVGGSLGGMFVLEWAYFGKDYVRCVVPIATSSRHSAWGISWGEAQRQSIYADPKYNDG
NCU07001.1	AAVIGGSLGGMFVLEWAYFGKDYVRCVVPIATSSRHSAWGISWGEAQRQSIYADPKYDDG
FG05658.1 	AAVVGGSLGGMFVLEWAYFGKDYIRCVVPIATSSRHSAWGISWGEAQRQSIYADPKYDDG
AN2229.1  	AAVVGGSMGGMLTLEYAYFGKDYVRAIVPIATSARHSAWCISWGEAQRQSIYSDPKYENG
          	***:***:***:.**:*******:*.:******:***** ************:****::*

MG01469.1 	YYPFSDPPSTGLGAARMSALLTYRSRNSFEARFGRNIPDPSRRQTIRERPRPSTPSEAHF
NCU07001.1	YYSFEDPPSTGLGAARMSALLTYRSRNSFEARFGRNTPDPSRRQIIRERPTPSTPSEAHF
FG05658.1 	YYSFDDPPSTGLGAARMSALLTYRSRNSFESRFGRNIPDPSKVQTIGGRPRPSTPSEAHF
AN2229.1  	YYSFDEPPAAGLGAARMSALLTYRSRNSFESRFGRNVPDPSKRQNINGTERLPTPPNEHW
          	**.*.:**::********************:***** ****: * *      .**.: *:

MG01469.1 	HIHNDGHNISRDGLSRHNSNGQGSKAANTGANPTATPAVAQAAAEERSMDPQFHGPEKKE
NCU07001.1	HIHNDGHKVKRTSLSRSNSNDSTGIAR-----PVDAPAASNASSHEAQSDPQFHGPQKTT
FG05658.1 	QIHNDGHVVKRTSISQSS--------------AADAASAPQTPAEPDVADPQFHGPSNGS
AN2229.1  	AIHNDGHKGNWSGR------------------NSPAPEKPAEKTEVQYMDPQFSGTKTFS
          	 ******  .  .                      :.  .   :.    **** *...  

MG01469.1 	-----SLTGGDAVP-TSTYFSAQSYLRYQGEKFVKRFDSNCYIAMTRKLDTHDVSRSRAP
NCU07001.1	TTTTTSLTGGDVMPPQSTYFSAQSYLRYQGSKFVKRFDSNCYIAMTRKLDTHDVSRGRAA
FG05658.1 	------LTGG-DMP-QSHYFSAQSYLRYQGRKFVTRFDSNCYIAMTRKLDTHDVSRNRAD
AN2229.1  	KS--VSTTDGNAQKRPATYFSAQSYLRYQGDKFVKRFDANCYIAITRKLDTHDVSRHRAR
          	.:   : *.*.     : ************ ***.***:*****:*********** ** 

MG01469.1 	S-----IANALAMIEQPTLVLGIESDGLFTFAEQEEIAEHVPNARLEKIDSPEGHDAFLL
NCU07001.1	S-----IAEALALIQQPTLVLGIESDGLFTFAEQEELAQHIPDSRLERIDSPEGHDAFLL
FG05658.1 	T-----IADALAMIQQPTLVLGIESDGLFTFAEQEEIAQHVPNARLERIESPEGHDAFLL
AN2229.1  	PDSENPVREALSQIQQPALVLGIESDGLFTFEEQKEIAEGIPDSRLKRIDSPEGHDAFLL
          	..:...: :**: *:**:************* **:*:*: :*::**::*:**********

MG01469.1 	QFEQVNRYTLEFLREVLPDIMEKDGDGSSETVESSVGQLTKSSTFGEAEVDDITAW
NCU07001.1	QFEQVNRYILNFLREVLPDIMNKDVPEGSEAAESSVGQITKSSTFGEAEVEDITAW
FG05658.1 	QFEQVNNYVLSFFKEVLPDIMSKD---GAVPEEASVGQITKSSTFGEAEVEDITAW
AN2229.1  	QFEQVNQYILEFFREVLPDIMSKTPTDGAAIDG--VGKLTKSSTFGEAEVEDITAW
          	******.* *.*::*******.*    .:      **::***********:*****
```
